# Spontaneous dissolution of a renal pelvic stone with intrastone gas: A case report with serial CT findings

**DOI:** 10.1016/j.radcr.2026.08.082

**Published:** 2026-09-25

**Authors:** Norio Yamamoto, Shinji Akao, Takesuke Yamashita, Kaoru Ono, Tsuneo Koganemaru

**Affiliations:** a Department of Urology, Tokuyama Medical Association Hospital, Yamaguchi, Japan; b Department of Surgery, Tokuyama Medical Association Hospital, Yamaguchi, Japan; c Internal Medicine, Yamashita Internal Medicine Clinic, Yamaguchi, Japan; d Internal Medicine, Ono Internal Medicine Clinic, Yamaguchi, Japan; e Department of Urology, Yuno Onsen Hospital, Yamaguchi, Japan

**Keywords:** Renal pelvic stone, Spontaneous dissolution, Intrastone gas, Computed tomography, Urolithiasis

## Abstract

Spontaneous dissolution of a large renal pelvic stone is extremely rare. We report the case of a 65-year-old man with paraplegia below Th6 who experienced recurrent febrile urinary tract infections beginning in May 2016. Noncontrast computed tomography (CT) in August 2016 revealed a right renal pelvic stone measuring 26 × 13 mm. During long-term follow-up, serial CT imaging demonstrated structural homogenization, central cavitation, progressive rim thinning associated with intrastone gas, and complete disappearance by November 2020. CT attenuation analysis suggested a uric acid–dominant core surrounded by a calcium-containing shell. Extremely low attenuation values within the central cavity (mean −364 HU) supported the presence of intrastone gas rather than fluid. These observations may suggest that dynamic interactions among urinary pH, stone composition, and intrastone bacterial activity may promote progressive internal weakening and spontaneous dissolution of mixed-composition renal stones.

## Introduction

Urinary tract stones are frequently associated with urinary tract infections (UTIs), and the 2 conditions often interact with each other. Alkalinization of urine caused by UTIs facilitates the formation of infection-related stones, whereas dehydration and urinary concentration lower urinary pH, creating a favorable environment for uric acid crystallization. In clinical practice, urinary pH varies depending on daily circumstances, and thus stones of mixed composition may develop. Therefore, strict categorization of urinary calculi into “infection-related” or “metabolic” types may warrant reconsideration.

With the widespread use of extracorporeal shock wave lithotripsy (ESWL) and advances in endourological techniques, minimally invasive stone fragmentation procedures have become the first-line treatment for renal calculi. However, with the growing elderly population, many high-risk patients with frailty or dementia cannot undergo active intervention and must be managed conservatively with symptomatic treatment and observation alone.

## Case presentation

A 65-year-old man had a history of pyogenic myelitis, resulting in thoracic spinal cord injury below the Th6 level since his forties. He had been hospitalized continuously for 23 years. His activities of daily living were severely limited: he was able to roll in bed with assistance, and transfer to a wheelchair required 2 caregivers. Urinary drainage was managed with a long-term indwelling urethral catheter.

Until April 2015, he experienced febrile episodes consistent with UTIs once or twice per year, which resolved promptly. Computed tomography (CT) examinations during that period revealed no hydronephrosis or urinary calculi.

From June to December 2016, however, he developed recurrent refractory fevers attributed to UTIs, with more than 10 episodes over 6 months. In August 2016, noncontrast CT revealed a right renal pelvic stone measuring 26 × 13 mm. A cavity-like appearance was noted in the upper portion of the stone (Fig. 1A). By November 2016, follow-up CT showed the cavity had disappeared, and the internal texture had become homogeneous (Fig. 1B). In March 2017, CT demonstrated onset of central dissolution (Fig. 2A). By November 2019, further central dissolution was evident, leaving only the peripheral rim (Fig. 2B). Finally, by November 2020, CT confirmed complete disappearance of the stone.

**Fig. 1:**
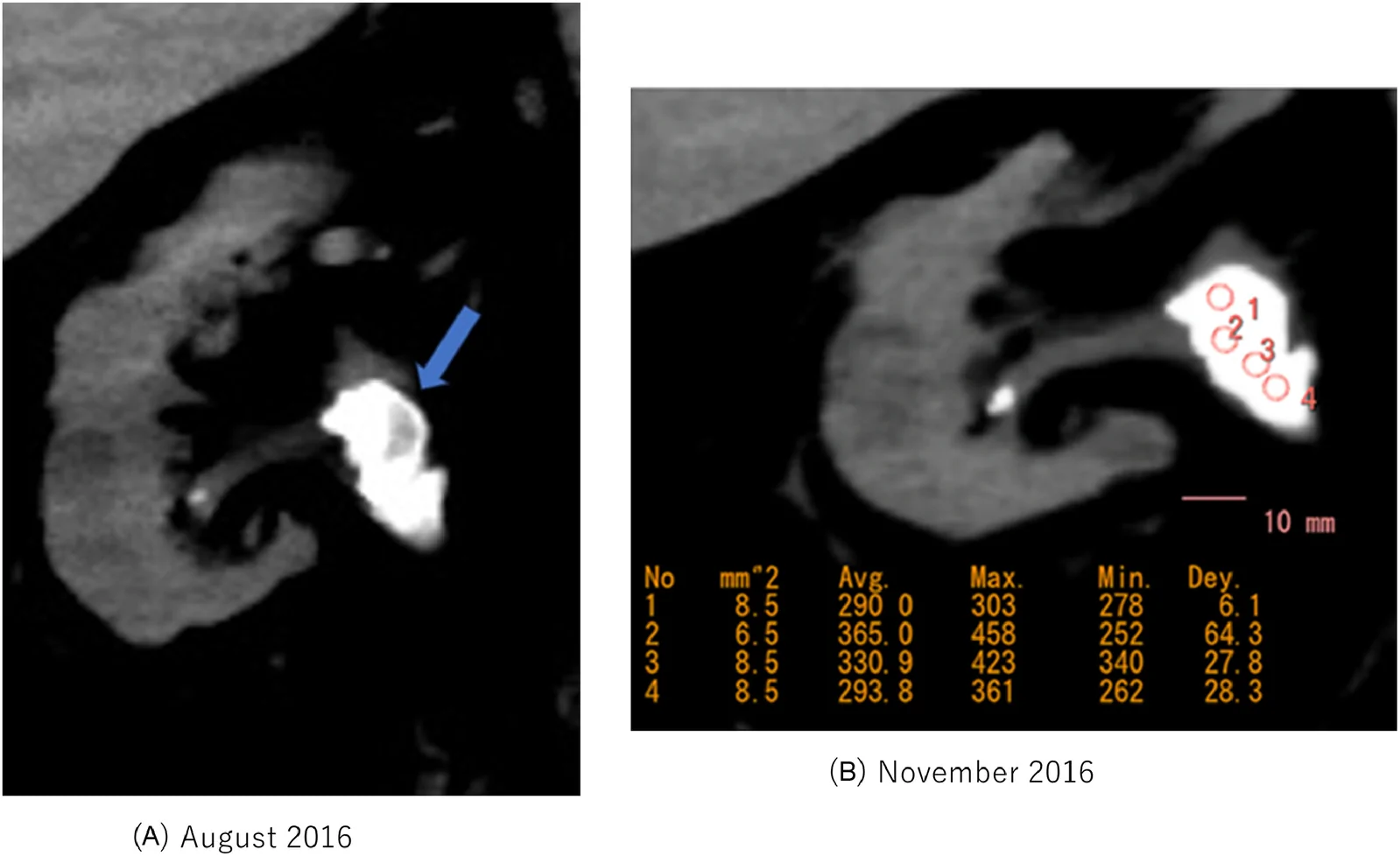
Initial formation and subsequent homogenization of the right renal pelvic stone on noncontrast CT. (A) August 2016: a small low-attenuation cavity (arrow) was observed in the upper portion of the stone. (B) November 2016: the cavity had disappeared and the internal appearance had become homogeneous. The mean CT attenuation value measured at 4 regions of interest (ROIs; 8.5 mm in diameter) was 320 HU (range, 290-365 HU). Scale bar = 10 mm.

**Fig. 2:**
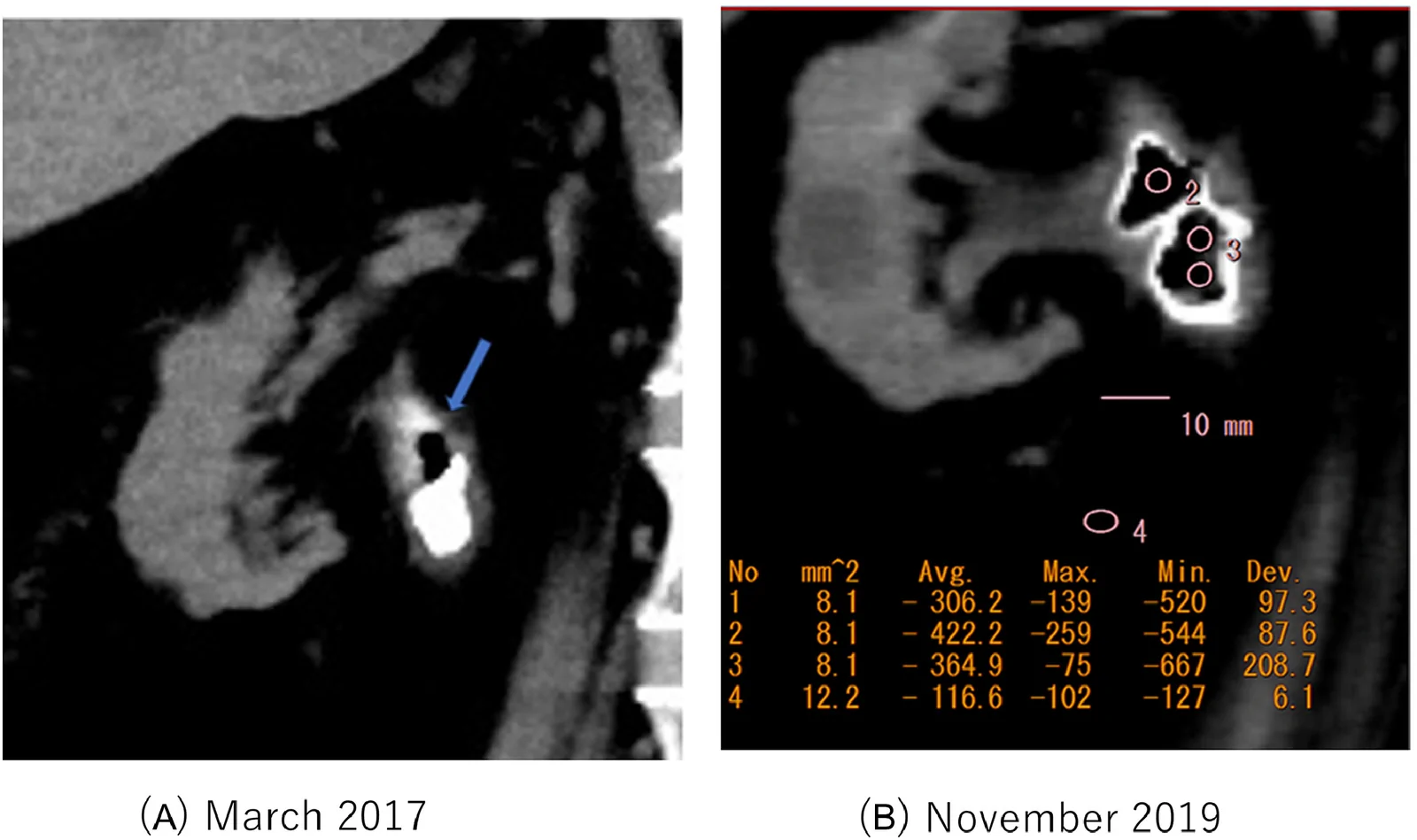
Sequential central dissolution and intrastone gas on noncontrast CT. (A) March 2017: partial dissolution had begun from the central region of the stone (arrow). (B) November 2019: further central dissolution with marked thinning of the peripheral rim and intrastone gas within the central cavity. The negative CT attenuation values measured within the cavity were consistent with gas. Scale bar = 10 mm.

The routine abdominal CT protocol used during the observation period employed a slice thickness of 4 mm, with images displayed at a window level of 50 HU and a window width of 250 HU.

## Discussion

Uric acid stones account for approximately 5%-10% of renal calculi and are strongly influenced by urinary pH [1]. During prolonged febrile illness, dehydration and metabolic stress may transiently acidify urine and promote uric acid crystallization. The present case provides a rare sequential CT record of stone formation, transformation, and eventual disappearance.

Kageyama et al. [2] measured CT attenuation values of 47 urinary stones and found that the values increased in the following order: uric acid stones (480 HU) < cystine stones (757 HU) < mixed calcium oxalate and calcium phosphate stones (1555 HU) [2].

CT attenuation values tend to be lower in vivo than in vitro. In this case, the initial mean attenuation of 320 HU (range 290-365 HU) is therefore compatible with an overall uric acid–dominant stone, and the centrally lucent area seen in Fig. 1A may represent a partially formed uric acid–dominant core. As the clinical environment later shifted toward alkalinization, the subsequent homogenization observed in Fig. 1B raises the possibility that this region became secondarily filled with ammonium urate sediment, resulting in a more homogeneous internal structure. Urinary pH values were retrospectively obtained from routine urinalyses performed during hospitalization. Urine specimens were generally collected as early-morning spot samples on the ward. Differences in urinary pH between the 2 periods were assessed using a 2-sample Student’s t-test assuming equal variances.

During the 6-month febrile period in 2016, urinary pH—which is typically elevated during UTIs—remained paradoxically low. As shown in Fig. 3, the mean urinary pH declined from 7.9 to 6.6 (*P* < .001), reaching 5.5 at the time of stone detection. Such sustained acidity promotes nucleation of undissociated uric acid (H_2_U), while the accompanying physiologic stresses—particularly dehydration, tissue catabolism, and reduced urine output—may hinder orderly crystal growth and structural consolidation. Urate salts (NaHU) may have encouraged heterogeneous deposition, providing a plausible basis for the transition from the heterogeneous appearance in Fig. 1A to the more uniform configuration observed later in Fig. 1B. By early 2017, the clinical environment had shifted substantially: febrile episodes had resolved, antibiotics were discontinued, and urinary pH rose steadily into the alkaline range.

**Fig. 3:**
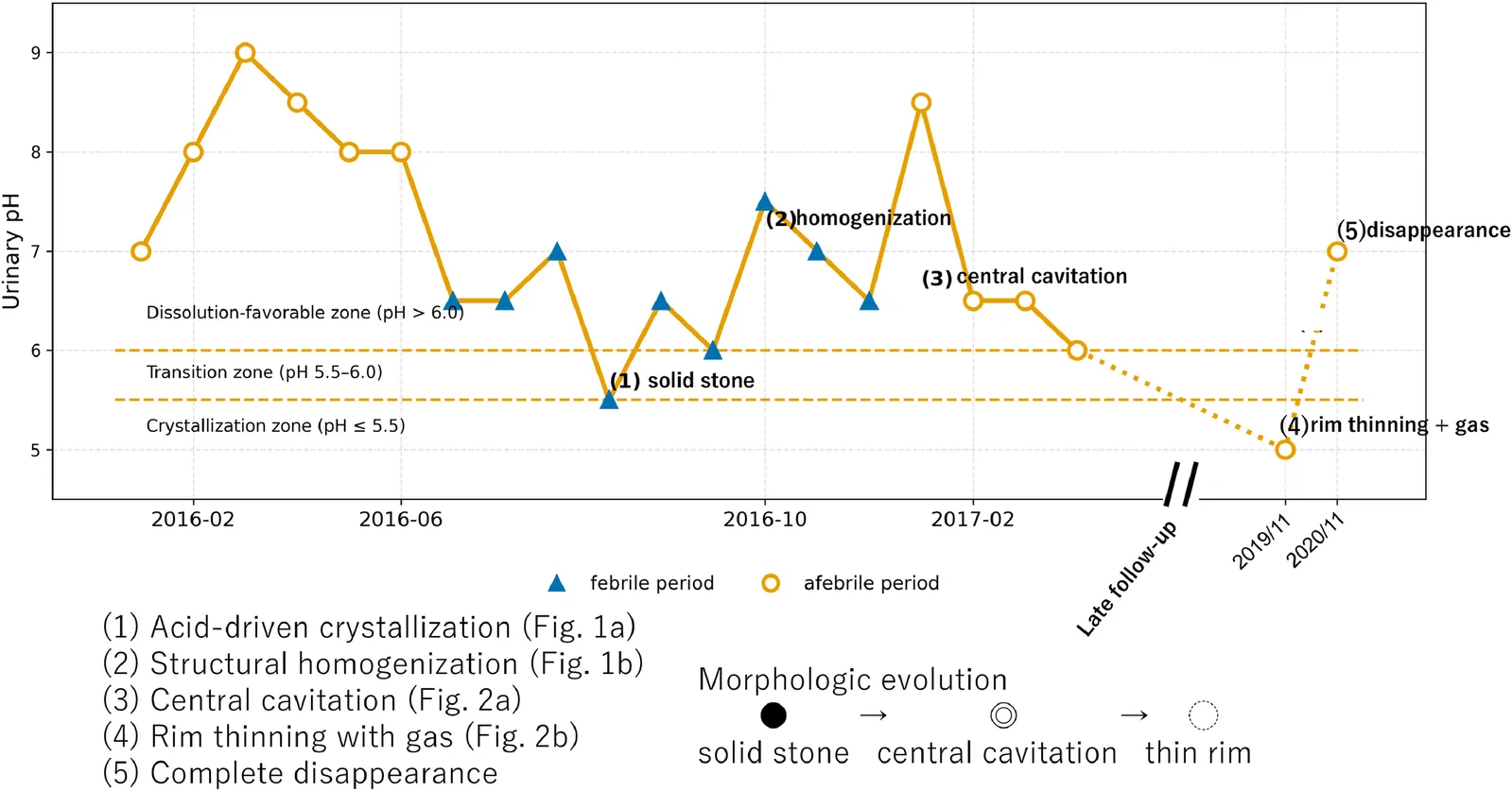
Integrated timeline of morphologic evolution and urinary pH changes. Serial CT findings are presented alongside longitudinal spot urinary pH measurements to illustrate the temporal relationship between changes in urinary pH and the morphologic evolution of the stone. The pH zones indicate general physicochemical conditions favorable for uric acid crystallization or dissolution and do not correspond directly to individual CT stages.

What initially appeared to be a single 26-mm stone later showed imaging features suggestive of 2 compositional elements after partial internal dissolution (Fig. 2A). The presence of an outer shell suggests a mixed structure, possibly incorporating calcium oxalate or carbonate apatite formed under infection-related conditions.

Urine cultures obtained repeatedly during the 2016 febrile period identified *Proteus mirabilis* in 6 samples and *Klebsiella* species in 2. This persistent condition raises the possibility that bacteria may have colonized microscopic fissures or pinhole-like channels penetrating the stone surface and persisted within the stone matrix.

In early 2017, after febrile episodes resolved and antibiotics were discontinued, intrastone bacterial metabolic activity may have resumed intermittently, potentially supported by nutrients supplied via urine penetrating through microfissures. Such activity offers a plausible, although unproven, explanation for the subsequent appearance of intrastone gas. Taken together, Fig. 4 presents a conceptual schematic of a proposed mechanism linking intrastone infection, gas formation, and progressive internal weakening of the stone. Support for this hypothesis is strengthened by the analysis of Takeuchi et al. [3], who examined 158 urinary calculi and reported that 102 were metabolic stones, whereas 56 were clearly infection-related stones. Notably, bacteria were cultured and identified from the stone matrix in more than 66% of these infection-related stones, indicating that bacteria can persist and survive within stones and function as a reservoir. CT imaging further reinforces this interpretation: the central dissolving cavity showed extremely low attenuation values, with a mean of −364 HU (range −306 to −422 HU), markedly lower than perirenal tissue (−116 HU), indicating substantial intrastone gas accumulation (Fig. 2B). Review of the entire CT series showed that gas was confined to the interior of the stone, with no gas identified elsewhere in the renal collecting system or renal parenchyma and no perinephric inflammatory changes. This degree of gas is difficult to explain by passive diffusion alone and may suggest that bacterial metabolic activity contributed to gas formation and internal weakening, although direct causation cannot be established.

**Fig. 4:**
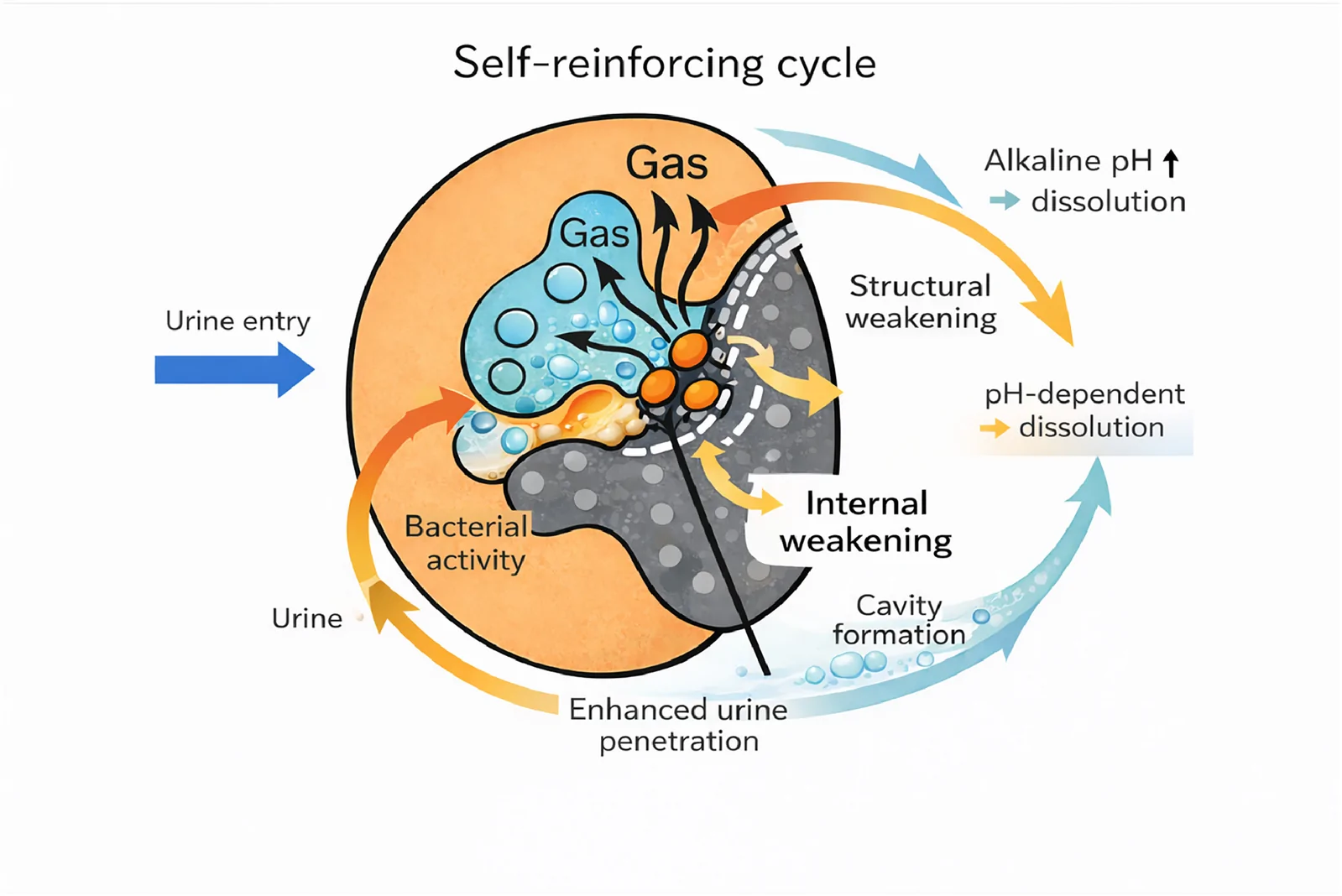
Conceptual illustration of a proposed mechanism for progressive stone dissolution and intrastone gas formation. This schematic illustrates a hypothetical self-reinforcing cycle in which urine penetration through fissures, bacterial activity, intrastone gas formation, structural weakening, and pH-dependent dissolution may have contributed to progressive central cavitation and eventual stone dissolution. This proposed mechanism is based on the longitudinal imaging and urinary findings in the present case and does not represent a proven causal pathway.

Although urinary pH had risen to 8.5 by early 2017, dissolution progressed from the center rather than the periphery. This atypical pattern is consistent with the proposed compositional architecture described above: a potentially more soluble uric acid–dominant core surrounded by a less soluble calcium-containing outer shell. The formation and dissolution of uric acid stones are strongly pH dependent. Undissociated uric acid (H_2_U) precipitates at pH values below approximately 5.5, whereas higher pH levels favor conversion to the more soluble species HU⁻ and subsequently to sodium urate (NaHU). Experimental work by Shimizu et al. [4] confirms that NaHU dissolves readily under alkaline conditions, providing a solid physicochemical basis for the progressive dissolution that occurred once urinary pH increased in 2017. Notably, these experimental data also suggest that monosodium urate may transiently recrystallize in the weakly acidic to neutral pH range before complete dissolution, which may account for the temporary homogenization of the intrastone cavity observed in Fig. 1B.

This sequential cascade of events—acid-driven crystallization during febrile episodes, structural homogenization of the stone interior, central cavitation, rim thinning with intrastone gas, and eventual disappearance—may provide a plausible mechanistic explanation for the stone’s natural history.

The complete disappearance of the stone without symptomatic passage may be explained by progressive thinning and internal weakening of the outer shell, accompanied by fragmentation of residual material until it became sufficiently small to pass silently or dissolve completely. Although several case reports have described spontaneous dissolution of renal stones, as summarized in Table 1 , most have provided only limited imaging information and lacked long-term serial CT evaluation, quantitative HU analysis, assessment of intrastone gas, or longitudinal urinary pH measurements. Among these reports, those documenting multiyear structural changes are particularly scarce, and multimodal integration of these parameters into a unified mechanistic interpretation has not been clearly demonstrated.

**Table 1:** Summary of previously reported cases of spontaneous renal stone resolution and comparison with the present case.

Study	Imaging modality and follow-up	Outcome/key findings	Difference from the present case
Elliot et al. [6]	Plain radiography only; follow-up for several months to years	Seventeen cases of spontaneous disappearance of renal stones	No serial CT, HU analysis, intrastone gas assessment, longitudinal urinary pH data, or structural evolution
Nguyen et al. [7]	CT; 3-week follow-up; drug-induced ureteral stone	Complete spontaneous dissolution	Ureteral stone; short-term CT follow-up; no intrastone gas.
Vellanki et al. [8]	Limited radiographic follow-up	Short-term radiographic changes in uric acid stones	No serial CT, quantitative HU analysis, or urinary pH time-series
Fahy et al. [9]	Ultrasound and plain radiography; pediatric case	Spontaneous resolution	Non-CT imaging only; no HU analysis, urinary pH monitoring, or structural data
Wazzan et al. [10]	CT; gas-containing staghorn stone	Intrastone gas associated with infection; treated with PCNL	No spontaneous dissolution or long-term serial CT follow-up
Hammad et al. [11]	CT; matrix stone with emphysematous pyelitis	Infection-related intrastone gas; delayed PCNL	Short-term imaging only; no spontaneous disappearance
Zawaideh et al. [12]	CT in 6 cases; literature review of 21 cases	Gas-containing renal stones; most required surgical intervention	No long-term serial CT evaluation and no documented spontaneous disappearance
Present case (Yamamoto et al.)	Serial noncontrast CT over 4 y; IVP; ultrasonography; longitudinal urinary pH measurements	Cavity formation → internal homogenization → rim thinning → complete disappearance	Long-term serial CT follow-up combined with intrastone gas assessment, HU analysis, and urinary pH time-series

The table summarizes imaging modalities, follow-up duration, key outcomes, and major differences from the present case.

From a clinical perspective, this case illustrates how dynamic interactions among urinary pH, infection activity, and stone microstructure may influence both the formation and subsequent dissolution of complex calculi. Profound acidity during febrile illness facilitated uric acid crystallization, whereas later transitions to alkaline urine enabled dissolution of the uric acid–rich core. In particular, longitudinal monitoring of urinary pH and infection status is important in patients who exhibit atypical radiologic features such as intrastone gas or central cavitation. The integration of CT morphology, quantitative HU changes, and biochemical trends in this patient demonstrates how combined clinical and imaging data can help clarify otherwise unexplained stone behavior and provides insight into the natural evolution and spontaneous dissolution of a renal pelvic stone.

Several limitations warrant consideration. Because this was a retrospective analysis of CT examinations obtained over several years, complete technical details of the original CT acquisition and reconstruction parameters were not available for all examinations.

Stone composition could not be confirmed directly because no fragments were retrieved, and dual-energy CT was not available at our institution during the observation period [5]. However, the relatively low CT attenuation values together with the clinical course are compatible with a uric acid–dominant stone. Furthermore, as a single case, the generalizability of the proposed mechanisms is inherently limited. Nevertheless, when considered alongside the literature summarized in Table 1, this case provides a highly detailed depiction of the natural course of a mixed-composition renal stone and suggests that spontaneous dissolution may occur in selected circumstances, particularly when biochemical and infectious conditions evolve favorably.

## Conclusion

We report a rare case of spontaneous dissolution of a renal pelvic stone documented by long-term serial CT imaging.

This case suggests that a stone with a proposed mixed composition, consisting of a uric acid–dominant core and a calcium-containing outer shell, may undergo stepwise structural transformation, including internal homogenization, central cavitation, and rim thinning associated with intrastone gas, ultimately resulting in complete disappearance. These findings may suggest that interactions among urinary pH, bacterial activity, and stone microstructure may play a critical role in the natural history of certain renal stones.

We report an unusual case in which serial CT imaging, attenuation measurements, and longitudinal urinary pH changes provided a detailed record of the spontaneous structural transformation and eventual disappearance of a renal pelvic stone.

## Ethical approval

This study was approved by the Institutional Review Board of Tokuyama Medical Association Hospital on November 14, 2025. The study was conducted in accordance with the Declaration of Helsinki.

## Patient consent

Written informed consent for publication was obtained from the patient (or from the patient’s legal guardian).
